# A Free App for Diagnosing Burnout (BurnOut App): Development Study

**DOI:** 10.2196/30094

**Published:** 2022-09-06

**Authors:** Jordi Godia, Marc Pifarré, Jordi Vilaplana, Francesc Solsona, Francesc Abella, Antoni Calvo, Anna Mitjans, Maria Pau Gonzalez-Olmedo

**Affiliations:** 1 Universitat de Lleida Lleida Spain; 2 IRBLleida Lleida Spain; 3 Fundacio Galatea Barcelona Spain

**Keywords:** diagnose burnout, Android app, medical informatics, health care, health professionals, mobile health, digital health, health applications, online health, mobile phone

## Abstract

**Background:**

Health specialists take care of us, but who takes care of them? These professionals are the most vulnerable to the increasingly common syndrome known as burnout. Burnout is a syndrome conceptualized as a result of chronic workplace stress that has not been successfully managed.

**Objective:**

This study aims to develop a useful app providing burnout self-diagnosis and tracking of burnout through a simple, intuitive, and user-friendly interface.

**Methods:**

We present the BurnOut app, an Android app developed using the Xamarin and MVVMCross platforms, which allows users to detect critical cases of psychological discomfort by implementing the Goldberg and Copenhagen Burnout Inventory tests.

**Results:**

The BurnOut app is robust, user-friendly, and efﬁcient. The good performance of the app was demonstrated by comparing its features with those of similar apps in the literature.

**Conclusions:**

The BurnOut app is very useful for health specialists or users, in general, to detect burnout early and track its evolution.

## Introduction

In this study, we deal with burnout syndrome. Burnout syndrome is becoming increasingly popular. It is not a disease but a signal of emotional distress. Significant efforts have been made to determine its causes.

In 1974, Freudenberger and Richelson [1] suggested that feelings of exhaustion, frustration, and tiredness are generated by an overload. He included the term work addiction in the explanation, also being the ﬁrst to propose that this type of relationship is associated with a productive imbalance. Freudenberger and Richelson [1] later expanded this theory by saying that these feelings were because of the irrational workloads imposed by the workers themselves or the people around them.

Maslach and Jackson [2] deﬁned burnout as a syndrome with three dimensions (the most widely accepted to date):

1. Emotional exhaustion: emotional exhaustion because of the demands of work
2. Depersonalization: indifference and apathy toward society
3. Low personal fulﬁllment: low feelings of success and personal fulﬁllment

Burnout is included in the 11th Revision of the International Classiﬁcation of Diseases [3] as an occupational phenomenon. It is not classiﬁed as a medical condition and is deﬁned in the 11th Revision of the International Classiﬁcation of Diseases as follows: burnout is a syndrome conceptualized as resulting from chronic workplace stress that has not been successfully managed. Burnout refers speciﬁcally to phenomena in the occupational context and should not be applied to describe experiences in other areas of life.

A partial list of potential contributing causes includes (1) length of training, (2) mentality of delayed gratiﬁcation, (3) insufﬁcient protected research time and funding, (4) long working hours, (5) imbalance between career and family, (6) hostile workplace environment, and (7) gender- and age-related issues [4]. Burnout can have a signiﬁcant negative impact on the quality of patient care by negatively inﬂuencing clinical decision-making, increasing medical errors and malpractice claims, and lowering patient satisfaction [5-7]. Burnout may also lead to high turnover, difﬁcult relationships between providers and staff, and drug and alcohol abuse [8].

In general, those most vulnerable to distress from the syndrome are professionals in whom worker-client human interactions of an intense or lasting nature are observed [9,10]. Balch and Shanafelt [11] found that health care professionals are at a disproportionately higher risk than other workers in stressful jobs that focus on public services. Burnout is markedly more common among physicians than depression, substance abuse, or suicide [11]. Shanafelt et al [12] reported that 45% of physicians had experienced at least one symptom of burnout. Another study found that high rates of depersonalization were the greatest among early-career physicians and decreased with age [13]. Burnout may affect >60% of family practice providers [14].

A recent study [15] found that physician turnover and reduced clinical hours attributable to burnout resulted in approximately US $4.6 billion in costs each year in the United States. The rising prevalence of burnout among physicians and other health care professionals has become a major policy concern in the United States during the COVID-19 pandemic [16]. Regardless of burnout status, the results showed that all professional health care groups had high levels of anxiety. Primary care physicians had signiﬁcantly higher anxiety scores than all other health care professionals. Thus, a sense of tension, anxiety, distress, and other symptoms of mental disorders [16,17] would greatly help detect burnout syndrome.

Preventive actions for burnout include checklists, tools for early detection, training programs for high-risk occupations, awareness-raising actions, and good practice guidelines [18]. Free and user-friendly apps could be good tools for tackling all the actions on the list, with guarantees of success.

In most of the research conducted on mobile apps available to detect burnout, such as MindDoc [19], Psychosomat [20], and BreathePro [21], no free tax and public algorithms were used, although they are very popular. By contrast, Lafraxo et al [22] created an app to detect burnout in a nursery where they used the Copenhagen Burnout Inventory (CBI) [23] algorithm.

The research question for this study is whether a free cloud-based mobile app provides potential patients with burnout syndrome with a diagnosis based on their needs and goals. This study presents a mobile app called BurnOut to offer potential patients with burnout syndrome a diagnosis generator based on their needs and goals using cloud-based mobile apps to help diagnose burnout using the CBI [23] and Goldberg Health Questionnaire (GHQ) [17], which are free and public algorithms. The use of these free and contrasted algorithms benefits the study as the final results can be followed up and reproduced for comparison.

In this study, we present the BurnOut app. Our proposal was focused on offering a self-operating tool to diagnose burnout. This also allows users to monitor their evolution. The most common instrument for diagnosing burnout is the licensed Maslach Burnout Inventory test, developed by Maslach and Jackson in 1981 [24]. The BurnOut app implements the CBI [23], a valid, free, and reliable alternative. It also implements a version of the GHQ with 12 questions used to detect mental disorders [17].

## Methods

### BurnOut App

**Figure 1:**
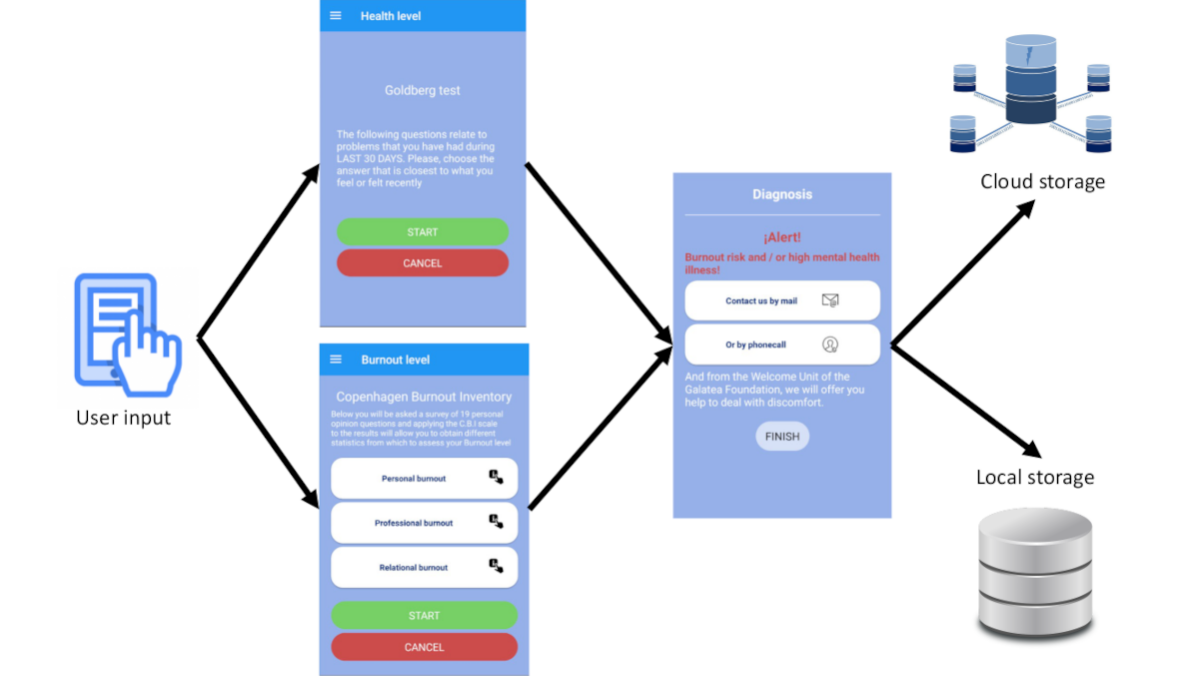
BurnOut app operation.

Figure 1 shows the basic operation of the BurnOut app. It represents the ﬂowchart between the user interface (UI) of the CBI and GHQ tests of the BurnOut app, which generates the ﬁnal custom diagnosis. The diagnosis is shown on the mobile display and saved jointly with the tests in the local database and cloud storage.

The BurnOut app provides 3 main features: data collection, burnout diagnosis, and user monitoring. The UI for such features is user-friendly. The app also provides language support in Spanish, Catalan, and English. The default language is the same as that used on the smartphone.

The app provides a mail contact for doubts or suggestions with the developer through an *About* section, where the user can check some informative data from the app as a legal copyright.

The Android version of the app can be downloaded from Google Play using the app link [25].

### User Testing

A sample of 40 random users (summarized in Table 1) representing different social levels, professional activities, ethnical proﬁles, ages, and genders was chosen from the cities of Lleida and Tarragona (Spain).

**Table 1:** Test participant features (N=40).

Feature and class		Participants, n (%)
**Gender**		
	Male	17 (43)
	Female	23 (58)
**Age (years)**		
	*<*30	30 (75)
	35-65	10 (25)
**Technological proﬁle**		
	Yes	13 (33)
	No	27 (68)

### Collecting User Data

The ﬁrst time a user signs up on the app, a questionnaire with the following information must be ﬁlled in:

1. Eating habits: a few standard questions about healthy eating habits such as the frequency of meals or the number of fruits and vegetables consumed per day
2. Physical activity: how often does one walk and whether they engage in moderate or intensive physical activity for at least 10 minutes; if so, the length and frequency are requested
3. Consumption of toxic substances: yes or no questions about consumption of drugs; if yes, consumption for the past 30 days is requested

These data provide information about the user’s lifestyle and factors that can cause psychosocial risk and affect their psychoemotional wellness.

### Tests

#### Overview

BurnOut uses 2 instrumentally proven tests to evaluate users’ burnout (with the CBI) and psychological discomfort (with the GHQ). The tests do not constitute a ﬁnal diagnosis but help give the professional an idea about their mental health and the perception of the risk of exhaustion, which can serve as a guide for a more precise diagnosis.

The main features of the GHQ and CBI are as follows:

1. GHQ: This test has several versions; the implemented version comprises 12 questions, used by the Medical College of Barcelona. It evaluates the presence or lack of psychological discomfort (ie, distress). GHQ has a binary score (true or false). Further details are provided in the GHQ Test section.
2. CBI: It comprises 19 multiple-choice questions to detect the symptoms of burnout. It has no ﬁnal score and evaluates 3 subscales: personal, work-related, and client-related burnout.

#### GHQ Test

The GHQ test evaluates the existence of psychological discomfort. It measures the sense of tension, depression, inability to defend oneself, anxiety-based insomnia, lack of self-conﬁdence and self-esteem, and other symptoms of mental disorders [16,17,26].

There are 4 variants of this questionnaire, and the GHQ-12 variant used in this study is recommended for measuring psychological distress. This test contains 12 questions (Textbox 1) about the emotional or psychological problems that the user experienced in the past 30 days. Each answer comprises 4 options that are equivalent to numerical values (Table 2). The bimodal scoring method was used in the BurnOut app by the ofﬁcial manual. The maximum score (number of points on the test) is 12, and the possible range is 0 to 12 [27]. A score ≥4 indicates the possible presence of mental distress, and a score ≥8 indicates the presence of various symptoms of stress-related psychological disorders. To ensure the diagnosis and avoid ignoring any symptoms, a score equal to or higher than the threshold value of 3 was classiﬁed as distress in the BurnOut app.

**Goldberg Health Questionnaire questions.**

**Questions**

1. Have you been able to concentrate well on what you did?
2. Have your worries made you lose a lot of sleep?
3. Have you felt that you play a useless role in life?
4. Have you felt capable of making decisions?
5. Have you felt under strain?
6. Have you ever felt that you cannot overcome your difﬁculties?
7. Have you been able to enjoy your activities every day?
8. Have you been able to deal adequately with your problems?
9. Have you felt unhappy and depressed?
10. Have you lost conﬁdence in yourself?
11. Have you thought that you are useless?
12. Do you feel reasonably happy, considering the circumstances?

**Table 2:** Goldberg Health Questionnaire answer values.

Question number and answer options			Value
**Question 1**			
	Better than usual	0	
	As usual	0	
	Less than usual	1	
	Much less than usual	1	
**Question** **2**			
	Not at all	0	
	No more than usual	0	
	A little more than usual	1	
	Much more than usual	1	
**Question** **3**			
	More useful than usual	0	
	As usual	0	
	Less than usual	1	
	Much less than usual	1	
**Question** **4**			
	More than usual	0	
	As usual	0	
	Less than usual	1	
	Much less than usual	1	
**Question** **5**			
	Not at all	0	
	No more than usual	0	
	A little more than usual	1	
	Much more than usual	1	
**Question** **6**			
	Not at all	0	
	No more than usual	0	
	A little more than usual	1	
	Much more than usual	1	
**Question** **7**			
	More than usual	0	
	As usual	0	
	Less than usual	1	
	Much less than usual	1	
**Question** **8**			
	More capable than usual	0	
	As usual	0	
	Less capable than usual	1	
	Much less capable than usual	1	
**Question** **9**			
	Not at all	0	
	No more than usual	0	
	A little more than usual	1	
	Much more than usual	1	
**Question** **10**			
	Not at all	0	
	No more than usual	0	
	A little more than usual	1	
	Much more than usual	1	
**Question** **11**			
	Not at all	0	
	No more than usual	0	
	A little more than usual	1	
	Much more than usual	1	
**Question** **12**			
	More than usual	0	
	Approximately the same as usual	0	
	Less than usual	1	
	Much less than usual	1	

#### CBI Test

The CBI is one of the most widely used burnout inventories [23,28]. The CBI questionnaire explores the following three dimensions of burnout:

1. Personal burnout: degree of fatigue or emotional exhaustion experienced by a person
2. Work-related burnout: Burnout related to one’s job, experienced in relation to the work without trying to establish causal relationships
3. Client-related burnout: the degree of emotional fatigue or exhaustion that someone experiences in relation to their work with other people

The psychometric qualities of this test make it a good tool for diagnosis and prevention. The CBI questionnaire is intended only to allow users to make a conservative self-assessment of their burnout status. This result is not intended as a medical diagnosis. However, it can inform them as to whether they should seek medical and psychotherapeutic assistance. Only a trained physician is qualiﬁed to advise on the initiation, modiﬁcation, or discontinuation of the medication.

The CBI comprises a survey of 19 questions that evaluate 3 dimensions that affect burnout symptoms (personal burnout [Textbox 2], work-related burnout [Textbox 3], and client-related burnout [Textbox 4]).

The questions in the CBI application did not appear in the same order, as shown here. The questions were mixed with those on other topics. This is recommended to avoid stereotypical response patterns. These dimensions comprise 3 independent subscales to determine the risk of burnout according to the combination of the scores.

These questions can have 2 different packs of 5 answers with a numerical value associated with them (Table 3). These packs are indistinctly used. There is only one exception in the 13th question of the work-related dimension—“Do you have enough energy for family and friends during leisure time?”— for which the score is reversed. The total score in the dimension is the average of the scores obtained in this dimension. Depending on this value, each dimension is categorized into one of the three burnout ranked levels: low, moderate, and high (Table 4).

The CBI ﬁnal diagnosis depends on the scores obtained for each dimension (Table 5).

**Copenhagen Burnout Inventory questions: personal burnout dimension.**

**Personal burnout questions**

1. How often do you feel tired?
2. How often are you physically exhausted?
3. How often are you emotionally exhausted?
4. How often do you think: “I can’t take it anymore”?
5. How often do you feel worn out?
6. How often do you feel weak and susceptible to illness?

**Copenhagen Burnout Inventory questions: work-related burnout dimension.**

**Work-related burnout questions**

1. Is your work emotionally exhausting?
2. Do you feel burned out because of your work?
3. Does your work frustrate you?
4. Do you feel worn out at the end of the working day?
5. Are you exhausted in the morning at the thought of another day at work?
6. Do you feel that every working hour is tiring for you?
7. Do you have enough energy for family and friends during leisure time?

**Copenhagen Burnout Inventory questions: client-related burnout dimension.**

**Client-related burnout questions**

1. Do you ﬁnd it hard to work with clients?
2. Do you ﬁnd it frustrating to work with clients?
3. Does it drain your energy to work with clients?
4. Do you give more than you get back when you work with clients?
5. Are you tired of working with clients?
6. Do you wonder how long you will continue working with clients?

**Table 3:** Copenhagen Burnout Inventory answer values.

Answer pack 1	Answer pack 2	Value
Always	To a very high degree	100
Often	To a high degree	75
Sometimes	Somewhat	50
Seldom	To a low degree	25
Never or almost never	To a very low degree	0

**Table 4:** Copenhagen Burnout Inventory dimension valuations.

Dimension	Level		
	Low	Moderate	High
Personal burnout	*<*50	50-74	75-100
Work-related burnout	*<*50	50-74	75-100
Client-related burnout	*<*50	50-74	75-100

**Table 5:** Copenhagen Burnout Inventory risk.

Copenhagen Burnout Inventory score	Cases
Low	Low-risk in all 3 dimensions; 2 low-risk dimensions and 1 moderate-risk dimension
Moderate	3 moderate-risk dimensions; 2 moderate-risk dimensions, 1 low-risk dimension, and 1 high-risk dimension
High	2 high-risk dimensions and 3 high-risk dimensions

### Diagnosis

**Figure 2:**
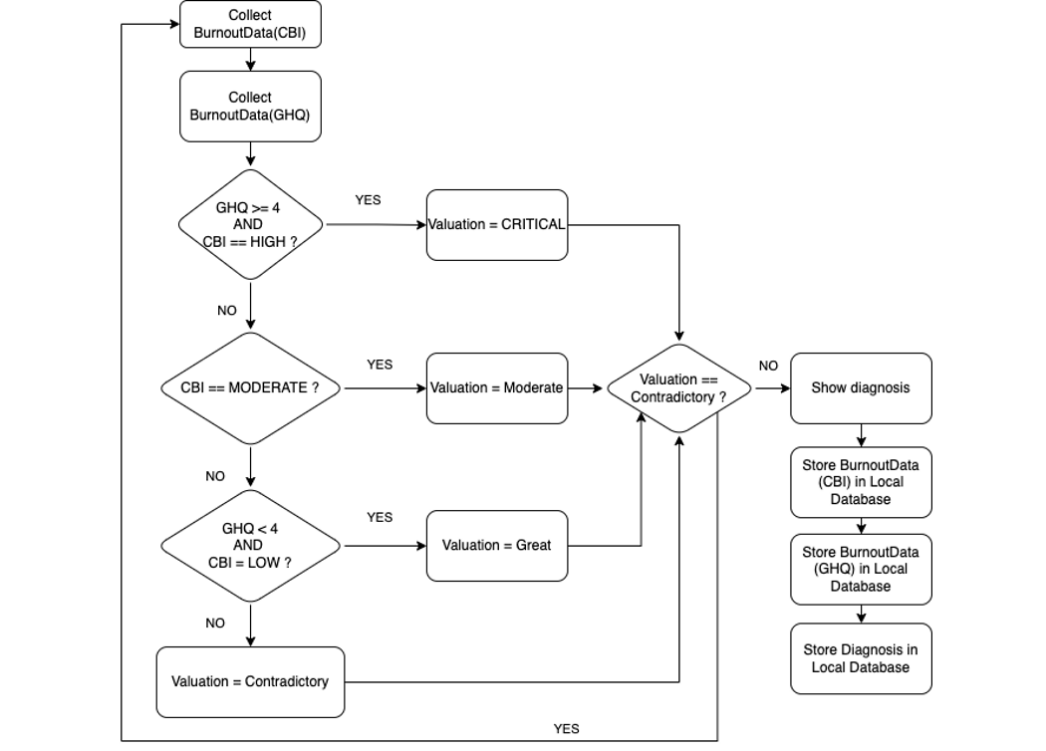
Diagnosis procedure. CBI: Copenhagen Burnout Inventory; GHQ: Goldberg Health Questionnaire.

Figure 2 explains how the BurnOut app obtains the diagnosis as a combination of GHQ (see the *GHQ Test* section) and CBI (see the *CBI Test* section) tests. There were four possible diagnoses:

1. Critical: high risk on the GHQ (the outcome is true) and high burnout risk on the CBI
2. Moderate: moderate burnout risk on the CBI (independently of the GHQ results)
3. Great: no risk on the GHQ and low burnout risk on the CBI
4. Contradictory: the app recommends repeating the tests

The resulting diagnosis is visualized and registered in the local database, as well as in the GHQ and CBI tests. The recommendation is to self-administer the tests and repeat them every 3 months.

### Monitoring

There are 3 ways of monitoring the evolution of the user’s health condition.

#### Burnout Stats Chart

The user can check the diagnosis of the past 4 CBI tests in a column chart.

#### Last Burnout Test Stats

A bar chart that hosts the last time the user passed the CBI test, indicating which state has improved or worsened to have a clearer view of the evolution and strengths and weaknesses. To increase the user’s feeling of doing well, when the dimensions are 0 after the last time they passed the test, instead of the chart, a message of congratulations appears to increase the user’s satisfaction, highlighting that the user is on the right path to wellness and encourages them to continue with the process.

#### Mental Health Indicator

If psychological discomfort is obtained in the GHQ test, a highlighted *Alert* message will appear to warn the user that they are in a critical state. If the state of mental health from the GHQ test diagnosis is deﬁned as wellness, a message of congratulations will appear to increase the user’s satisfaction. This will encourage them to continue the process. In other words, it will increase adherence to using the BurnOut app.

### BurnOut App Implementation

We attempted to follow guidelines to ensure a pleasant design and an easy-to-use app. The reason is that iOS mobile personal health record apps have better usability scores than Android apps, as stated in the study by Zapata et al [29], after analyzing a wide range of apps.

Currently, there are many alternative technologies to develop apps for mobile devices. Developing the app twice, in the native code for Android and iOS, implies extra production costs. Furthermore, the use of open-source tools for creating cross-platform apps using HTML, Cascading Style Sheets, and JavaScript with a framework such as *PhoneGap* could decrease performance and lead to a lack of advanced device-speciﬁc features provided by the latest application programming interfaces (APIs).

The BurnOut app was developed using an efﬁcient approach—*Xamarin*, a Microsoft cross-platform framework that offers native performance and API access to provide a native user experience.

Xamarin allows the use of Model-View-ViewModel (MVVM) pattern, which implies making a clean separation between the logic and UI. This implies that Android and iOS will use the same code on the logic side, called core (shared logic code), leading to faster development by reducing duplicated code. When modifying the code, it is not essential to change the logic on both platforms—only from the speciﬁc *View*. Then, speciﬁc platform *Views* must be developed using native code for both Android and iOS. The framework provides methods to bind the UI with the core, and it will be automatically updated when a core property is changed.

Owing to the MVVM pattern, there is a clean separation among the View, which is the UI shown to the user on the screen; the Model, which contains the data of the current view; and the View-Model, which handles the communication between the view and the model.

The business and validation logic is performed just once as they are included in the core. This method reduces the coding as the core is shared between the platforms.

Thus, the BurnOut app is written using native API access and native performance and has easy maintainability while providing faster development.

However, the Xamarin framework and MVVM patterns have some drawbacks. As it is designed for cross-platform apps, unnecessary libraries are loaded at the app start-up, resulting in a noticeably slow process and an increase in the required memory size.

### Local Database

The BurnOut app uses 2 storage systems: a NoSQL local database, which is used by both Android and iOS, and *Akavache*, an asynchronous, persistent key-value storage system.

### Cloud Synchronization

The BurnOut app provides cloud synchronization among multiple devices, achieved by a server that implements a representational state transfer service with Spring. Representational state transfer has quickly become the de facto standard for building services on the web as it is easy to build and consume. In addition, it provides application security (encryption and authentication). Caching is built into the protocol. Service routing through the domain name system is a resilient and well-known system that is ubiquitously supported.

Cloud storage is implemented following the same technique. It stores the key-value registries on a server. Cloud data are asynchronously synchronized in the background so that the synchronization process is almost unnoticeable to the user. It performs incremental backup; therefore, it only uploads new or modiﬁed data. When users sign in, the local database is updated according to the data stored in the cloud. When a user signs out, the local database is erased as it would no longer be used and would already be saved in the cloud. This avoids inconsistencies between the local database and the cloud.

## Results

### Overview

In this section, the robustness, usability, and efﬁciency of the BurnOut app are tested.

To rigorously evaluate the application robustness, we recorded all the crashes that occur when using the app to control where and when they occur.

To measure usability, people had to perform several actions within the app, such as registering, introducing data, testing diagnoses, and monitoring tools. They also rated the user experience of the BurnOut app between 1 and 5. In addition, changes made based on their observations helped make the BurnOut app more user-friendly.

The efﬁciency of the BurnOut app is compared with that of Android and iOS devices in Table 6. The efﬁciency parameters measured were start-up, diagnosis generation, and log-in. Start-up is deﬁned as the elapsed time for an app to start. Diagnosis generation is the elapsed time the app spends generating a personalized diagnosis plan for the user’s needs. Log-in is deﬁned as the time required for the app to log the user in and set up their health status for monitoring and fetching the data from the cloud or database. These times were the averages of 3 different measurements. Times <1 second guarantee that the user’s train of thought remains uninterrupted [30].

**Table 6:** Devices used to test the Burnout app efficiency.

Operating system and device		Version
**Android**		
	Xiaomi Redmi Note 8	Android 10
	Xiaomi Redmi Note 5	Android 9
	Samsung Galaxy S4	Android 5.0.2
	Samsung Galaxy J3	Android 5.1
	LG G2	Android 4.4
**iOS**		
	iPhone X	iOS 14
	iPhone 8	iOS 11
	iPhone 6 Plus	iOS 10.3
	iPhone 6	iOS 10.3
	iPhone 5	iOS 10.3

### Robustness

A total of 17 crashes were detected during a testing period of 15 days of using BurnOut. Most of them occurred when loading data from the local database asynchronously (3/17, 18%), loading data from the cloud database (11/17, 65%; considering that it is still under implementation), and logging in (4/17, 24%). Furthermore, 11% (2/17) of them were because of simple programming errors, such as null pointer exception, memory allocation, and communication between app and server, and others were produced by database exceptions because of bad queries and mistreatment of asynchronous behavior.

### Usability

The ﬁrst users who evaluated the BurnOut app were positive toward it and, in general, thought that its usability was good. Some encountered issues that were mentioned in the study by McIlroy et al [31] regarding problems when reviewing mobile apps.

To measure the usability of the BurnOut app, a short survey of the users mentioned in the *User Testing* was section conducted using the industry-standard System Usability Scale (SUS) [32]. It comprises a 10-item questionnaire with 5 response options ranging from strongly agree to strongly disagree. It enables the evaluation of a wide variety of products and services, including hardware, software, mobile devices, websites, and applications. The SUS was chosen as its characteristics ﬁt our interests perfectly: it is a very easy scale to administer to participants, it can be used on small sample sizes with reliable results, and it is valid (ie, it can effectively differentiate between usable and unusable systems).

The participants ranked each question from 1 to 5 based on how much they agreed with the statement they were reading. A score of 5 meant they agreed completely, and a score of 1 meant they disagreed vehemently. All the testers answered the survey. The group had representative proportions of age, sex, and physical condition compared with the complete treatment group. Table 7 presents the obtained SUS results.

The rationale behind the calculation is very intuitive. The total score is 100, and each question weighs 10 points.

As odd-numbered questions are all in a positive tone, if the response is *strongly agree*, they are given the maximum score, which is 10 for each question. If the response is *strongly disagree*, they are given the minimum score, which is 0. By subtracting 1 from each of the odd-numbered questions, we ensure that the minimum is 0. Then, by multiplying by 2.5, we ensure that the maximum is 10 for each question.

In contrast, if the response is *strongly agree* for the even-numbered questions in a negative tone, they are given the minimum score, which is 0 for each question. If the response is *strongly disagree*, they are given the minimum score, which is 0. Thus, by subtracting the points for each question from 5, we ensure that the minimum is 0. Then, by multiplying by 2.5, we ensure the maximum is 10 for each of the questions.

Once all the results have been obtained, we calculate the average value for each test, thus obtaining the ﬁnal score. In this case, we obtained a score of 95.8. These results are incredibly satisfying as, according to the general guideline for the interpretation of an SUS score (Table 8), we achieved an excellent rating.

The results obtained were consistent with the sensations shared by the users after performing the tests. All of them considered that the web part was very intuitive and did not require much time to learn how to get the most out of it, whereas all of them highlighted the simplicity-usefulness relationship of the bot.

**Table 7:** System Usability Scale survey with 40 randomly selected users in Lleida and Tarragona.

Questions	1	2	3	4	5
I think that I would like to use this system frequently	0	0	0	7	33
I found the system unnecessarily complex	40	0	0	0	0
I thought the system was easy to use	0	0	0	11	29
I think that I would need the support of a technical person to be able to use this system	30	10	0	0	0
I found the various functions in this system were well integrated	0	0	0	16	24
I thought there was too much inconsistency in this system	40	0	0	0	0
I would imagine that most people would learn to use this system very quickly	0	0	0	3	37
I found the system very cumbersome to use	34	6	0	0	0
I felt very conﬁdent using the system	0	0	2	8	30
I needed to learn a lot of things before I could get going with this system	35	5	0	0	0

**Table 8:** Interpretation of System Usability Scale score.

System Usability Scale score	Rating
>80.3	Excellent
68-80.3	Good
68	Okay
51-68	Poor
<51	Awful

### Efﬁciency

First, the start-up efﬁciency was evaluated (Multimedia Appendix 1). The ﬁrst thing to point out here is that using Xamarin.Forms alongside the MVVMCross framework instead of using native languages added a noticeable delay when starting up the app on both Android and iOS. This is because it usually loads several libraries.

Multimedia Appendix 2 shows the measured time taken to obtain a diagnosis on both platforms. A performance analogous to that obtained by the start-up was obtained. In general, the Android and iOS outcomes were very similar.

Multimedia Appendix 3 shows the diagnosis loading, although retrieving it from the local database once it is cached. The BurnOut app performs slightly faster on iOS than on Android. In absolute terms, this represents a difference of 0.48 seconds.

In addition, the time difference between the best and the worst result when generating the diagnosis was 0.656 seconds and 0.652 seconds on Android and iOS, respectively. Overall, we can assure that the iOS version was slightly faster than the Android version.

When comparing diagnosis loading from the local database on the user progress section, Android was slightly faster than iOS by just 0.434 milliseconds; however, on older devices, the difference was very signiﬁcant. For example, iPhone 5 was 1.6 times faster than LG G2 when performing the same task.

As a concluding remark, BurnOut app operations are ﬂuent with high response times because of the implementation of asynchronous tasks to compensate for the slowness of the Xamarin framework. According to the results, we can asseverate that the performance difference between Android and iOS is very low.

## Discussion

### Principal Findings

The BurnOut app is a useful, user-friendly app that provides the most accurate possible diagnosis approach, focusing on the psychosocial risks that cause burnout syndrome according to the CBI and monitoring user evolution over time in a cross-platform system with interesting extras such as mental health evaluation through the GHQ.

The BurnOut app offers the main functionalities that a potential patient of burnout syndrome may need, as shown in the *Results* section.

The BurnOut app is robust and user-friendly as the users who took the test had an SUS score of 95.8 out of 100, which qualifies as excellent.

Regarding start-up efﬁciency, the app was noticeably slower than the app using native tools (not shown in this paper). This, through the use of Xamarin, is because of some extra libraries being added, which took some time to load; however, this is strictly necessary as it allows us to use MVVMCross framework to save a lot of time by sharing some code and the ability to ﬁx any bug once instead of ﬁxing it on each platform. In general, the Android and iOS outcomes regarding start-up when using Xamarin were very similar.

In contrast, it is not easy to compare the performance with the other apps listed in Table 9, as each app offers different features. None of them generates a custom diagnosis based on CBI and GHQ results, which is one of the main advantages of our app.

In addition, the BurnOut app uses a local database and will soon use cloud storage. This means that data would never be lost because of mobile malfunctions as the data would be constantly synchronized and stored in the cloud. Upload and download operations would take at most 3 to 5 seconds. In addition, synchronization, as well as local storage in the database, would be performed asynchronously in the background to avoid poor user experience.

Some of the apps shown in Table 9 do not have synchronization in the cloud and therefore do not communicate with any server. Thus, waiting times were almost eliminated. However, time penalties in the BurnOut app are imperceptible to the users, and we can assure them that they will never lose information as it is saved in a server cloud.

**Table 9:** BurnOut app market popularity.

App	Downloads (thousands)	Ratings	Average rating	Pro version
BurnOut app	N/A^a^	N/A	N/A	No
MindDoc	>3,000,000	37,341	4.4	Yes
Psychosomat	>10,000	117	4.3	Yes
Breathe Pro	>100,000	662	4.5	Yes

^a^N/A: not applicable.

### Comparison With Prior Work

The available tools to measure burnout among health care professionals have various strengths and limitations. Most health care systems will be able to ﬁnd a validated instrument or instruments that meet their particular needs and situations [33]. Table 10 summarizes 7 common tests in terms of their overall strengths and limitations. The most commonly used instrument to measure burnout among health care professionals is the Maslach Burnout Inventory [30]. It comprises 3 domains: emotional exhaustion, depersonalization, and a low sense of personal accomplishment. Other instruments available to measure burnout include the Oldenburg Burnout Inventory [34] and CBI [23]. The Oldenburg Burnout Inventory has 3 domains: physical, cognitive, and affective exhaustion and disengagement from work. The CBI has 3 domains: personal (physical and psychological fatigue and exhaustion), work (physical and psychological fatigue and exhaustion related to work), and client-related (or a similar term such as patient and student) burnout. Some health systems and investigators use the Physician Worklife Survey single item (“Overall, based on your deﬁnition of burnout, how would you rate your level of burnout?”) to measure burnout symptoms [35].

A wide range of preventive actions was reported by Eurofound’s correspondents, from awareness-raising activities such as information campaigns to training, consultation with health professionals, sharing examples of good practices, and the provision of tools to conduct risk assessments on stress and early detection of burnout [18,36].

The US National Academy of Medicine published a valid and reliable list of instruments for measuring burnout [37]. Each tool has its advantages and drawbacks, and some are more appropriate for speciﬁc populations or settings.

Let us examine the burnout app market. *Moodpath*, *Psychosomat,* and *BreathePro* are the most popular Android apps. *MindDoc* is also available for iOS.

*Moodpath* provides support for depression, psychotherapy, and mental health. It is based on a 2-week depression screening. It functions as a mood diary. It provides a list of the symptoms detected in the diary. It provides the user’s mental health assessment and helps understand the psychology behind the user’s mood. *Psychosomat* provides support for depression, including on the basis of burnout syndrome. Support is provided before, during, and after psychotherapy, as well as in self-discovery processes. Owing to the user-deﬁned criteria, it is also suitable for bipolar disorders. *BreathePro* is a kind of breathing training program. It helps avoid burnout and stress in users and measures stress resistance through an iPhone camera.

Jung et al [38] found that customer ratings were more critical to the survival of free apps, and there was also a beneﬁt from entering the markets early. Various app store analyses were presented in the study by Martin et al [39]. A strong correlation between rating and downloads (popularity) and the fact that free apps have higher ratings than nonfree apps were the most interesting ﬁndings. Tian et al [40] studied 1492 high- and low-rated apps from Google Play. They concluded that the size of the app, number of promotional images on the store page, and target Software Development Kit version are the features that most accurately differentiate apps with high ratings from those with low ratings.

Table 9 presents a comparison between the burnout apps according to downloads, ratings (how many users have evaluated that app), average rating (0-5), and the availability of its Pro version. Table 11 shows a comparison between the main features provided by the burnout apps.

All the apps except the BurnOut app provide similar features and share the same weakness. All of them focus on offering the monitoring of symptoms.

Another important concluding remark of this analysis is that overall, the analyzed apps do not offer any kind of interactivity or feedback between the patients and the clinicians.

The BurnOut app is the only app that supports burnout test diagnosis. It uses the CBI and GHQ tests. CBI and GHQ are royalty free; hence, their use is free. This is a significant advantage when the goal is to develop a free app.

**Table 10:** Strengths and limitations of burnout measures [33].

Test	Strengths	Limitations
MBI^a^ 22 items	Strong psychometrics; robust data showing scores correlated with outcomes of interest; can detect meaningful effect sixes from interventions	Cost; length; moderately complex to analyze; may not be sensitive to change over a short time frame
MBI 2 items	Strong psychometrics; short; robust data showing scores correlated with outcomes of interest	Cost; may not be sensitive to change
CBI^b^ 16 items	May be used by all professionals; free	Length; moderately complex to analyze; limited data showing scores correlated with outcomes of interest among health care professionals in the United States
OBI^c^ 19 items	May be used by all professionals; free	Length; moderately complex to analyze; limited data showing scores correlate with outcomes of interest
PWS^d^ 1 item	Short; free; simple to analyze; may be used by all professionals	Length; limited data showing scores correlated with outcomes of interest; too brief to have strong psychometrics; limited emotional exhaustion domain of burnout

^a^MBI: Maslach Burnout Inventory.

^b^CBI: Copenhagen Burnout Inventory.

^c^OBI: Oldenburg Burnout Inventory.

^d^PWS: Physician Worklife Survey.

**Table 11:** Burnout app features.

App	User-friendly interface^a^	External widget support	Cloud synchronization	Depression support	Burnout diagnosis	Mental health diagnosis	Customized assessment (based on results)	Personal contact (human support)
BurnOut	Yes	No	Yes	No	Yes	Yes	Yes	No
MindDoc	Yes	No	Yes	Yes	Yes	Yes	Yes	No
Psychosomat	Yes	No	No	Yes	No	No	No	No
Breathe Pro	Yes	No	No	No	Yes	No	No	No

^a^According to the Google guidelines stated in Google Design Guidelines.

### Limitations

The BurnOut app was not evaluated in a clinical trial with a balanced cohort comprising an intervention group of real diagnosed patients with burnout and another control group of people without this syndrome. The difﬁculty is to collect reliable results. Potential patients should access the app and obtain a diagnosis. This makes it very difficult to obtain satisfactory results.

### Conclusions

The BurnOut app provides potential patients with burnout syndrome with a diagnosis generator based on their needs and goals. It is also important to understand the contextual use of the app and how it affects communication services [41]. This study analyzes the most popular operating systems and device approaches and provides an overview of the possible frameworks for building a multiplatform app. In addition, the BurnOut app integrates native iOS and Android services to provide a powerful but native feel and look.

Both the iOS and Android versions of the BurnOut app were implemented using Xamarin, a Microsoft framework. It was proven that the BurnOut app is robust. The Android and iOS versions were compared in terms of several key points. The efﬁciency on both platforms was tested, resulting in very similar performance.

The user experience was enhanced by using native APIs. This hides the fact that a multiplatform framework was used to build the app.

Overall, the usability (with a 95.8 SUS score), efficiency, and robustness of the app were good enough, as the people who were surveyed had no problems when using the app, and most of them felt confident (Table 7). We know that users are willing to use personal health records [42], and it has been demonstrated that it can help users reach their goals successfully while keeping track of their health data and assisting them to follow a personalized plan that will fulﬁll their needs perfectly and help improve their wellness.

Future trends could focus on the implementation of notiﬁcations to keep users motivated during long working days and encourage them. These notiﬁcation messages and tips and the frequency with which they are sent would depend on the user’s registered results and health state. In addition, it could be used to remind them to continue using the app and encourage them to continue with the process.

As the View-Model logic is the same across platforms such as Android, iOS, or the web, an interesting new feature would be porting the app to the web to make it easy for the users to access all their web-based data.

## Appendix

Multimedia Appendix 1 Start-up efﬁciency (in seconds).

Multimedia Appendix 2 Diagnosis generation efﬁciency (in seconds).

Multimedia Appendix 3 Local stats loading for tracking user progress efﬁciency (in seconds).

## Abbreviations

API: application programming interface
CBI: Copenhagen Burnout Inventory
GHQ: Goldberg Health Questionnaire
MVVM: Model-View-ViewModel
SUS: System Usability Scale
UI: user interface
