# Supplementary figures and images for: A Free App for Diagnosing Burnout (BurnOut App): Development Study
Source: JMIR Med Inform. 2022 Sep 6;10(9):e30094. doi: 10.2196/30094 (PMC9490524; doi:10.2196/30094)

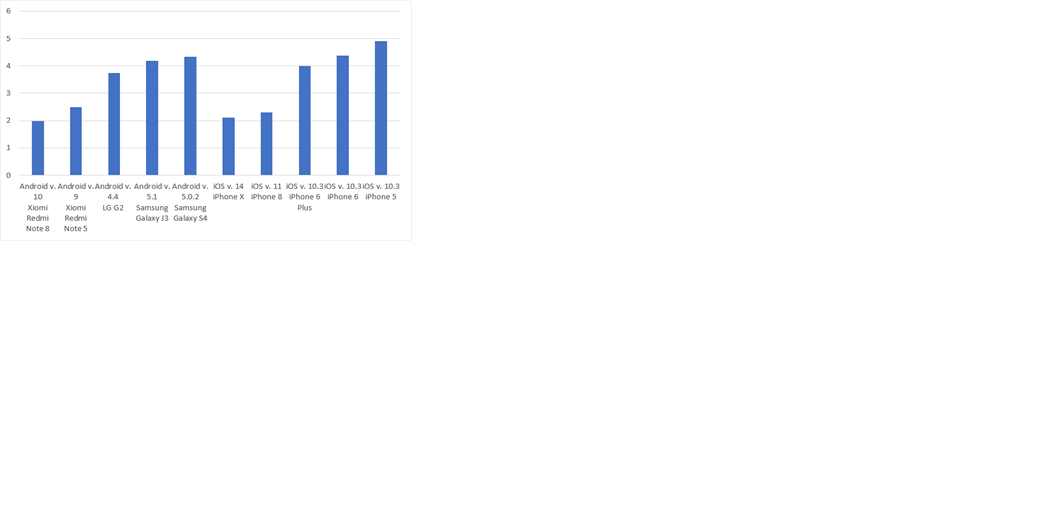

Supplement: Multimedia Appendix 1 [file medinform_v10i9e30094_app1.png]

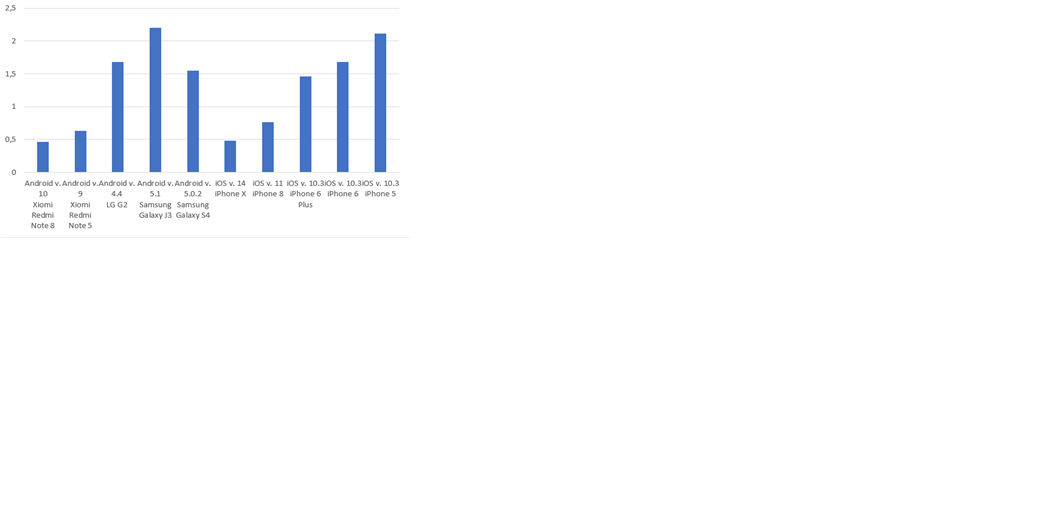

Supplement: Multimedia Appendix 2 [file medinform_v10i9e30094_app2.png]

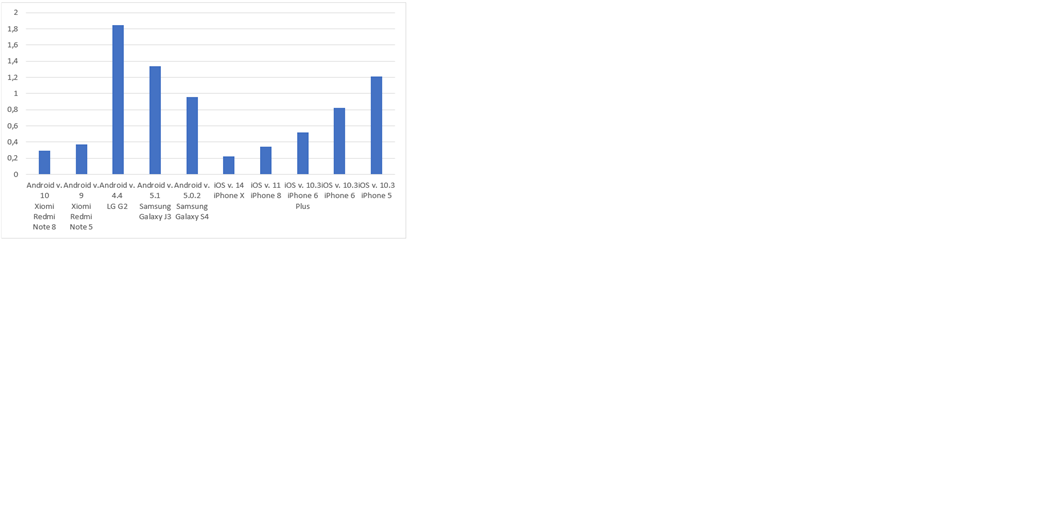

Supplement: Multimedia Appendix 3 [file medinform_v10i9e30094_app3.png]
